# Chemical, Antioxidant and Toxicological Profile of the Tropical Marine Sponges *Suberites aurantiacus*, *Mycale angulosa* and Halichondrida from Brazil

**DOI:** 10.3390/md24090305

**Published:** 2026-08-31

**Authors:** Nathália Cristina Lopes de Jorge, Paula Ivani Medeiros dos Santos, Renata Mendonça Araujo, Jorge Anderson Nascimento dos Santos, João Vinícius Soares Rocha, Leonardo de Medeiros Aquino, Hugo Alexandre Oliveira Rocha, Raquel Cordeiro Theodoro, Geórggia Fátima Silva Naliato, Elizeu Antunes dos Santos

**Affiliations:** 1 Laboratório de Química e Função de Proteínas Bioativas, Departamento de Bioquímica, Centro de Biociências, Universidade Federal do Rio Grande do Norte, Natal 59072-970, RN, Brazil; paulaims@gmail.com (P.I.M.d.S.); jorge.anderson.107@ufrn.edu.br (J.A.N.d.S.); jviniciussr9@gmail.com (J.V.S.R.); 2 Laboratório de Microbiologia do Instituto de Medicina Tropical (IMT-UFRN), Natal 59077-080, RN, Brazil; raquel.theodoro@ufrn.br (R.C.T.);; 3 Instituto Federal de Educação, Ciência e Tecnologia do Rio Grande do Norte—Campus Macau, Macau 59500-000, RN, Brazil; 4 Instituto de Química, Universidade Federal do Rio Grande do Norte, Natal 59078-970, RN, Brazil; renata.mendonca@ufrn.br; 5 Laboratório de Biotecnologia de Polímeros Naturais (BIOPOL), Departamento de Bioquímica, Centro de Biociências, Universidade Federal do Rio Grande do Norte, Natal 59072-970, RN, Brazil; leonardo.demedeiros.aquino@gmail.com (L.d.M.A.); hugo-alexandre@uol.com.br (H.A.O.R.)

**Keywords:** antioxidant compounds, porifera, secondary metabolites, tropical sponges, toxicology

## Abstract

The Potiguar Basin, in Rio Grande do Norte (Northeastern Brazil), has an extensive coastline with a rich diversity of marine sponges that remain poorly explored for their biotechnological potential. In this study, three sponge species—*Suberites aurantiacus*, *Mycale (Zygomycale) angulosa*, and Halichondrida—were collected, and a preliminary extraction was then performed to remove the most hydrophobic fraction with *n*-hexane. The remaining sediment was re-extracted with chloroform and methanol (1:1), and the resulting extracts (ESUB, EMYC, and EHALI, respectively) were analyzed. Chemical characterization using Gas–Liquid Chromatography–Mass Spectrometry (GC/MS) revealed distinct profiles for each genus, with the presence of alcohols, alkaloids, phenolic compounds, and lipids, mainly sterols. ESUB presented 17 identified compounds, EMYC had 15 identified and EHALI showed 15 identified. The antioxidant potential of the extracts was evaluated in vitro using DPPH radical scavenging, copper ion chelation, and total antioxidant capacity (TAC). The EC_50_ values (mg/mL) for DPPH were 0.05 (ESUB), 0.14 (EMYC), and 0.06 (EHALI), while for copper chelation they were 0.03, 0.034, and 0.026, respectively. Preliminary toxicological assays indicated low toxicity, with IC_50_ values above 9.6 mg/mL in human erythrocytes and *Tenebrio molitor* larvae, demonstrating a favorable safety profile for potential therapeutic applications. The study presents a preliminary screening of underexplored species in an equally underexplored region and supports further studies on their bioactive potential.

## 1. Introduction

Oxidative stress is directly associated with aging and with the development of diseases such as cancer, Alzheimer’s disease, Parkinson’s disease, diabetes, and chronic inflammation [1]. Chemical modifications in DNA, including base oxidation and strand breaks, generate mutations that increase the risk of cancer development; when accumulated over time, these alterations contribute to cellular aging, mainly by accelerating telomere shortening and dysfunction [2].

ROS can also induce lipid peroxidation in cellular membranes, triggering a cascade of biochemical reactions that may ultimately lead to cell death, while proteins may undergo structural and functional damage through chemical and conformational modifications [3]. In addition, ROS influence cellular signaling pathways and can determine the specific mechanisms of cell death involved, including apoptosis, ferroptosis, autophagy, and necroptosis [4]. Within this framework, molecules derived from marine sponge extracts may play an important role in oxidative regulation.

Approximately 15,000 sponge species have been described, and more than 5000 compounds have been isolated from these organisms, representing nearly 30% of the natural products derived from marine organisms with reported biological activities [5]. For this reason, sponges are considered promising sources for the discovery and development of new pharmaceuticals.

Historically, the first demonstration of the pharmaceutical potential of marine-derived products is attributed to the work of Bergmann and Feeney in 1951, involving a marine sponge [6]. The Caribbean sponge *Tectitethya crypta* yielded the nucleosides spongothymidine and spongouridine, which subsequently served as the structural basis for the development of the chemotherapeutic agent Cytarabine, widely used in the treatment of several forms of leukemia [7].

Among the primary metabolites isolated from marine sponges with reported biological activities, several studies can be highlighted. Polysaccharides obtained from the sponge *Erylus discophorus* exhibited potent anti-HIV activity [8]. Likewise, saturated and unsaturated fatty acids present in extracts from marine sponges of the genus *Latrunculia* were associated with antioxidant activities observed in vitro [9]. Similar findings were reported for fatty acids obtained from *Tetilla rodriguesi*, which demonstrated both antioxidant and antimicrobial activity against bacterial and fungal species [10].

In the search for bioactive compounds, tropical regions are particularly relevant because they harbor the greatest diversity of sponge species, making them a rich source of such metabolites [11]. Nevertheless, although several studies conducted in Brazil have explored the potential of marine sponges, the northeastern region of the country remains relatively underexplored [12,13,14]. Indeed, large portions of certain hydrographic basins have received little attention regarding the chemical characterization and biological evaluation of compounds derived from marine sponges.

Brazil possesses approximately 8000 km of coastline that remains largely unexplored with respect to the discovery of marine natural products [13]. Within this context, a specific coastal region of northeastern Brazil deserves particular attention: the Potiguar Basin.

The Potiguar Basin exhibits remarkable biodiversity. In 2008, 68 marine sponge species were reported in this region between the municipalities of Galinhos and Areia Branca. Additionally, approximately 40 other species were not included in that publication, suggesting that the actual diversity of these organisms may be substantially higher. Among the 68 species described, 25 were already recognized for their biotechnological relevance, including antimicrobial activities reported for 17 species. For the remaining 45 species for which no biological activity had yet been assigned, further studies may lead to the identification of novel bioactive compounds. Consequently, the sponges inhabiting this basin can be considered of considerable economic and biotechnological potential [15].

The investigation of extracts obtained from sponges inhabiting this coastline therefore represents a unique opportunity for the discovery of bioactive compounds with diverse biological activities. In this context, the present study aimed to expand the chemical knowledge of marine sponge species occurring in this region by collecting three different species—*Suberites aurantiacus*, *Mycale (Zygomycale) angulosa*, and representatives of the order Halichondrida—and subjecting their extracts to chemical characterization using gas–liquid chromatography coupled to mass spectrometry (GC–MS), as well as evaluating their antioxidant potential and preliminary toxicological safety through both in vitro and in vivo assays.

## 2. Results

### 2.1. Extract Yield

In the present study, three crude extracts were obtained from three species of marine sponges collected in the Potiguar Basin along the coast of the state of Rio Grande do Norte, Brazil. The extracts were designated according to the sponge species as follows: ESUB for *Suberites aurantiacus*, EMYC for *Mycale angulosa*, and EHALI for representatives of the order Halichondrida. Each extract (Table 1) was obtained from 200 g (wet weight) of sponge biomass. The resulting crude extracts were subsequently subjected to chemical characterization by gas–liquid chromatography coupled with mass spectrometry (GC–MS).

### 2.2. Chemical Characterization of the Extracts

In order to obtain a broader chemical characterization, the crude extracts were not fractionated prior to analysis, thereby avoiding potential compound losses that could occur during separation into subclasses. Chemical characterization performed by gas–liquid chromatography coupled with mass spectrometry (GC–MS) enables the detection of volatile compounds and molecules with relatively hydrophobic characteristics. For the extracts analysed, metabolites belonging to the classes of alkaloids, alcohols, phenolic compounds, and lipids were identified, with sterols and fatty acids—both belonging to the lipid class—representing the predominant compounds.

From this analysis, 17 compounds were identified in the extract obtained from *Suberites aurantiacus*. Among these, cholestanol (63.43%) represented more than half of the total analysed sample, as shown in Table 2.

The following table summarizes the compounds detected in ESUB and their respective chemical classes (Table 3):

For the extract obtained from *Mycale angulosa* (EMYC, Table 4), a total chromatographic coverage of 94.54% of the sample area was achieved, corresponding to 15 identified compounds. The predominant constituents were cholestan-3-ol (27.68%), dodecyl acrylate (21.75%), and cholesterol (15.17%), respectively.

The following table illustrates the compounds identified in EMYC and their corresponding chemical classes (Table 5).

For the extract obtained from sponges belonging to the order Halichondrida (EHALI, Table 6), 15 compounds were identified, corresponding to 97.40% of the total chromatographic area of the sample. The major constituents were ergosta-5,24(28)-dien-3-ol (3β) (19.33%) and cholesterol (17.56%).

The following table summarizes the compounds detected in EHALI and their respective chemical classes (Table 7).

### 2.3. Antioxidant Potential

**Figure 1:**
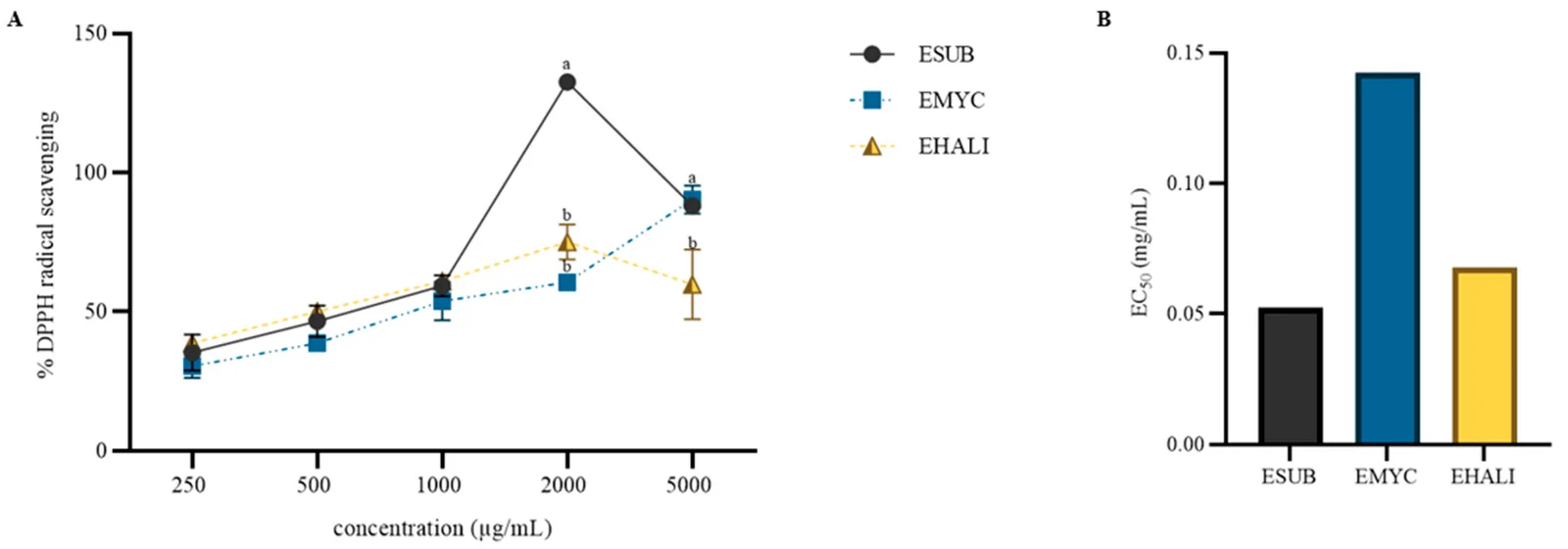
(**A**) Percentage of DPPH radical scavenging activity for the three sponge extracts at different concentrations. (**B**) Corresponding EC_50_ values. Data are presented as the mean of triplicates ± standard deviation. Different letters (a, b) indicate statistically significant differences among extracts at the same concentration. Statistical comparisons were performed using Analysis of Variance followed by Tukey’s test and Dunnett’s test.

All extracts exhibited dose-dependent DPPH radical scavenging activity, indicating that antioxidant capacity increased as extract concentration increased. Regarding the highest activity peak observed, the extract ESUB demonstrated a pronounced activity of 132% (± 0.91) at a concentration of 2000 µg/mL, surpassing the other extracts at all tested concentrations. However, a reduction in activity was observed at the highest tested concentration (5000 µg/mL) (Figure 1).

The extract EMYC was the only sample in which a clear dose-dependent activity was observed up to the highest tested concentration, with scavenging values of 30.38% (± 1.67), 38.73% (± 1.19), 53.78% (± 1.06), 60.55% (± 0.76), and 90.35% (± 2.44), respectively.

**Figure 2:**
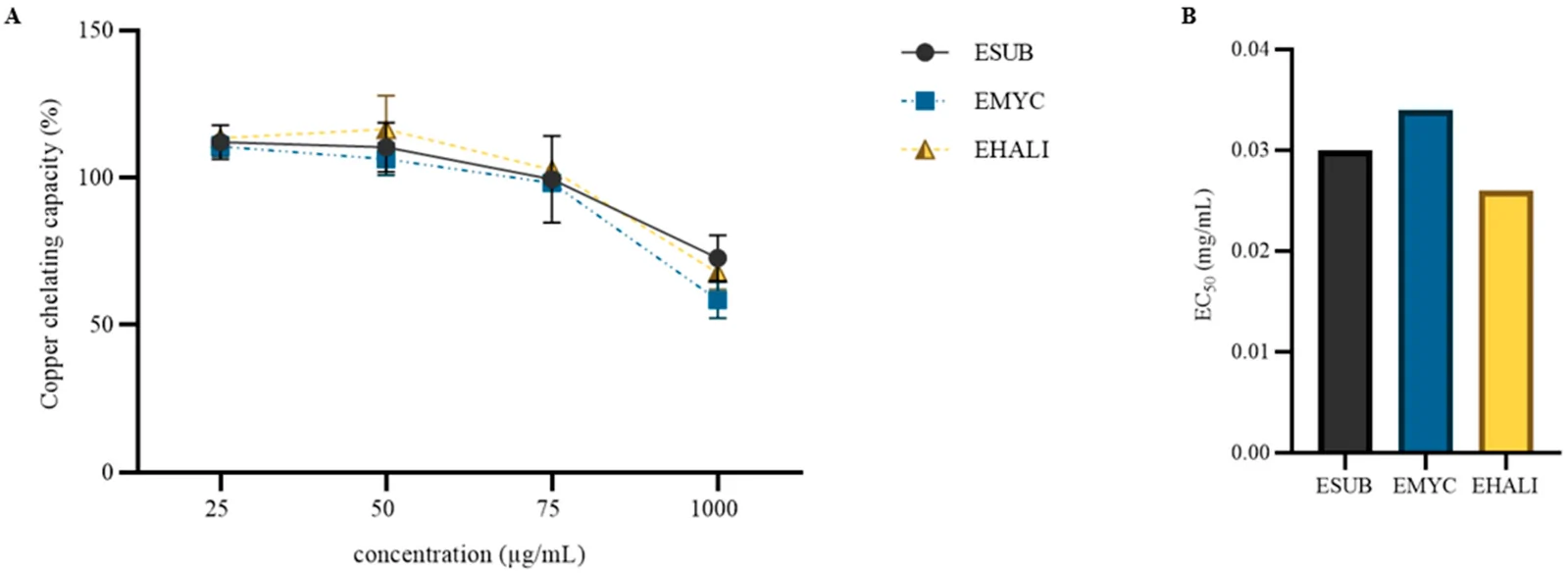
(**A**) Percentage of copper-chelating capacity for the three extracts at different concentrations. (**B**) Corresponding EC_50_ values. Data are expressed as the mean of triplicates ± standard deviation and were analyzed using Analysis of Variance followed by Tukey’s test and Dunnett’s test (*p* < 0.05).

The extracts also exhibited copper-chelating activity (Figure 2). The extract EHALI showed the highest chelating capacity (113.45% ± 1.93), followed by ESUB (112.1% ± 1.64) and EMYC (110.6% ± 1.02). No statistically significant differences were observed either among the different concentrations within each sample or when comparing the chelating activity among the three extracts. It is important to note that, due to the analytical method employed, the graph representing the percentage of copper chelation exhibits a decline in activity at higher concentrations. This effect results from sample precipitation at concentrations above 1000 µg/mL, which may interfere with spectrophotometric readings.

**Figure 3:**
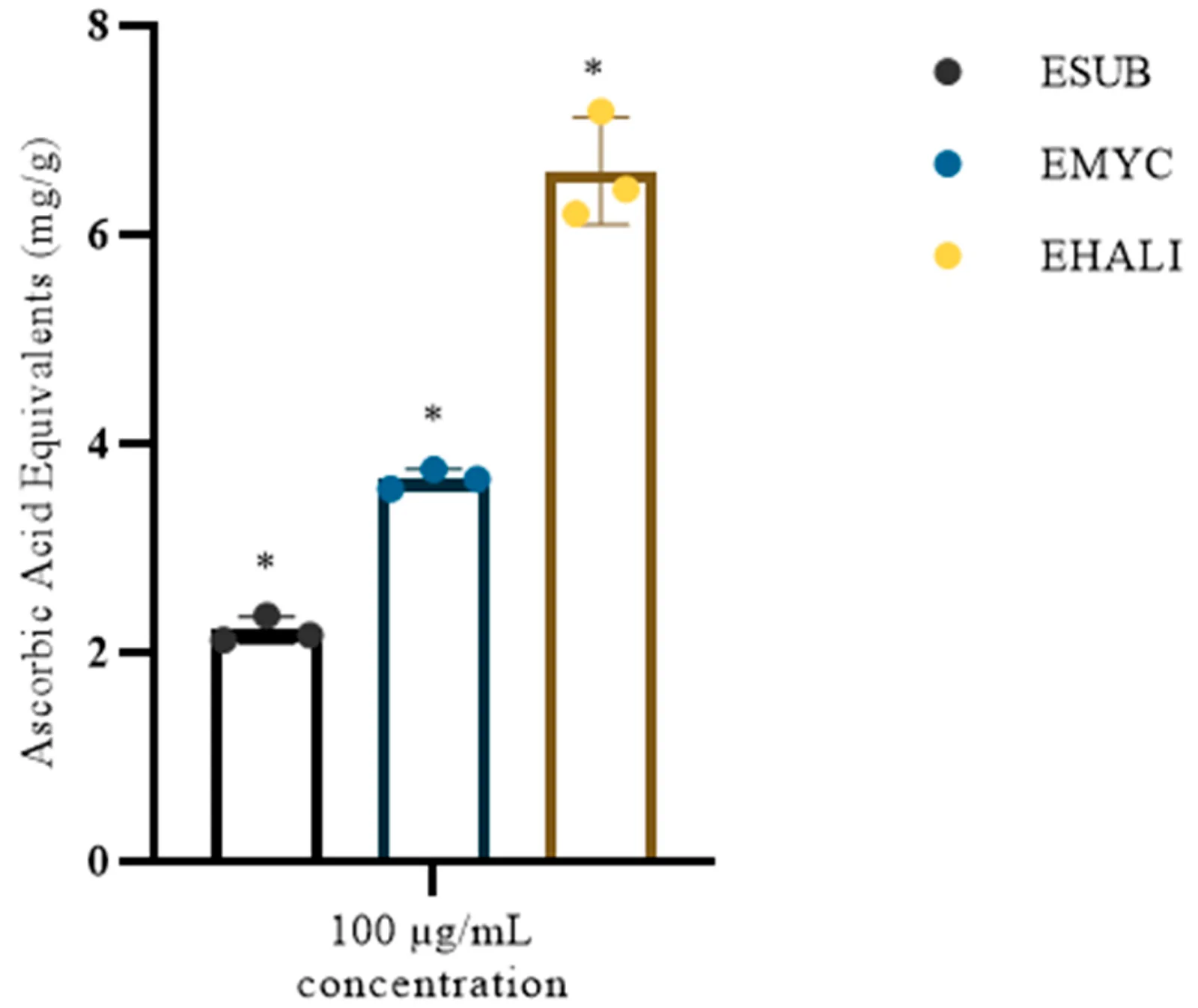
Total antioxidant capacity of the extracts expressed as ascorbic acid equivalents (mg/g). The dots represent the triplicates of each sample. The asterisk (*) indicates the presence of a statistically significant difference (*p* < 0.0001) among the extracts. Data are presented as the mean of triplicates ± standard deviation and were analyzed using Analysis of Variance followed by Tukey’s test and Dunnett’s test.

Next, the total antioxidant capacity of the extracts was evaluated. Among the tested samples, EHALI exhibited the highest activity, with a TAC value of 6.6 mg ascorbic acid equivalents per gram of sample (Figure 3).

### 2.4. Toxicity

**Figure 4:**
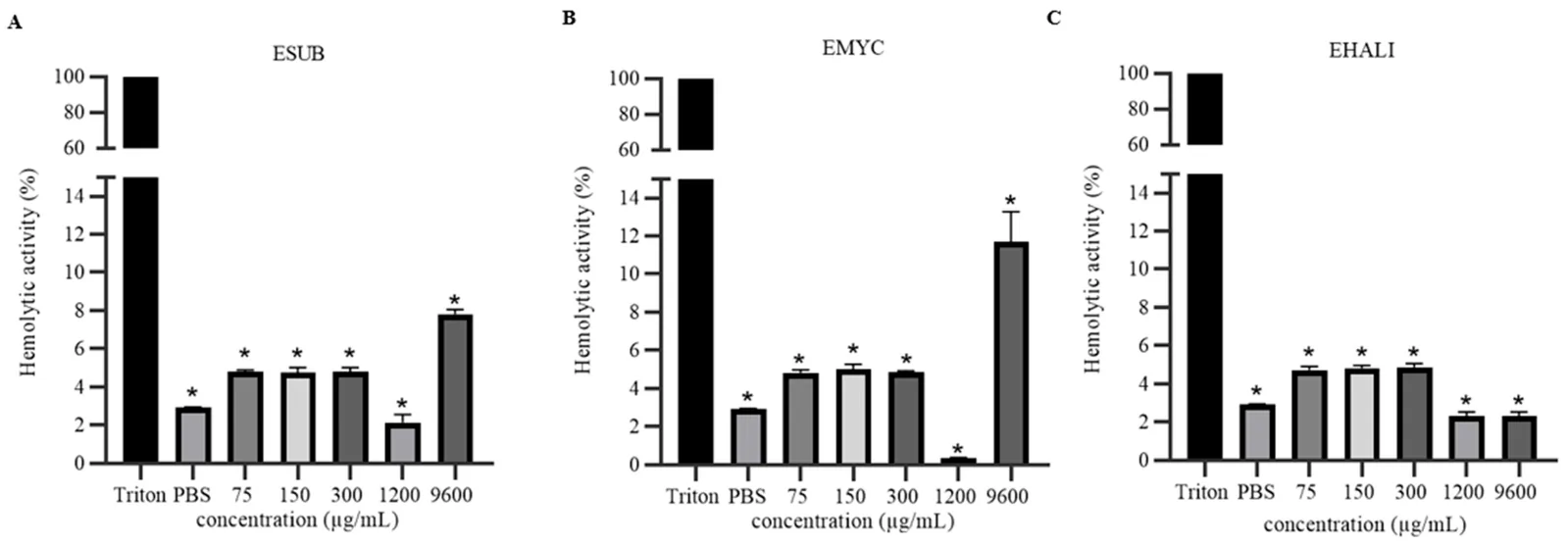
Hemolytic activity of the three sponge extracts ((**A**) ESUB; (**B**) EMYC; (**C**) EHALI). Red blood cells treated with 1% Triton X-100 were used as the positive control for hemolysis, whereas the negative control consisted of erythrocytes treated with Phosphate-buffered saline. Values are expressed as the mean of triplicates ± standard deviation. * *p* < 0.05 relative to the positive control.

The toxicity of the extracts was evaluated at different concentrations (75, 150, 300, 1200, and 9600 µg/mL) using in vitro assays with human erythrocytes and in vivo assays employing larvae of *Tenebrio molitor*. The results indicated that none of the extracts exhibited cytotoxic activity against erythrocytes at any of the tested concentrations when compared with the positive hemolysis control, 1% Triton X-100 (Figure 4).

**Figure 5:**
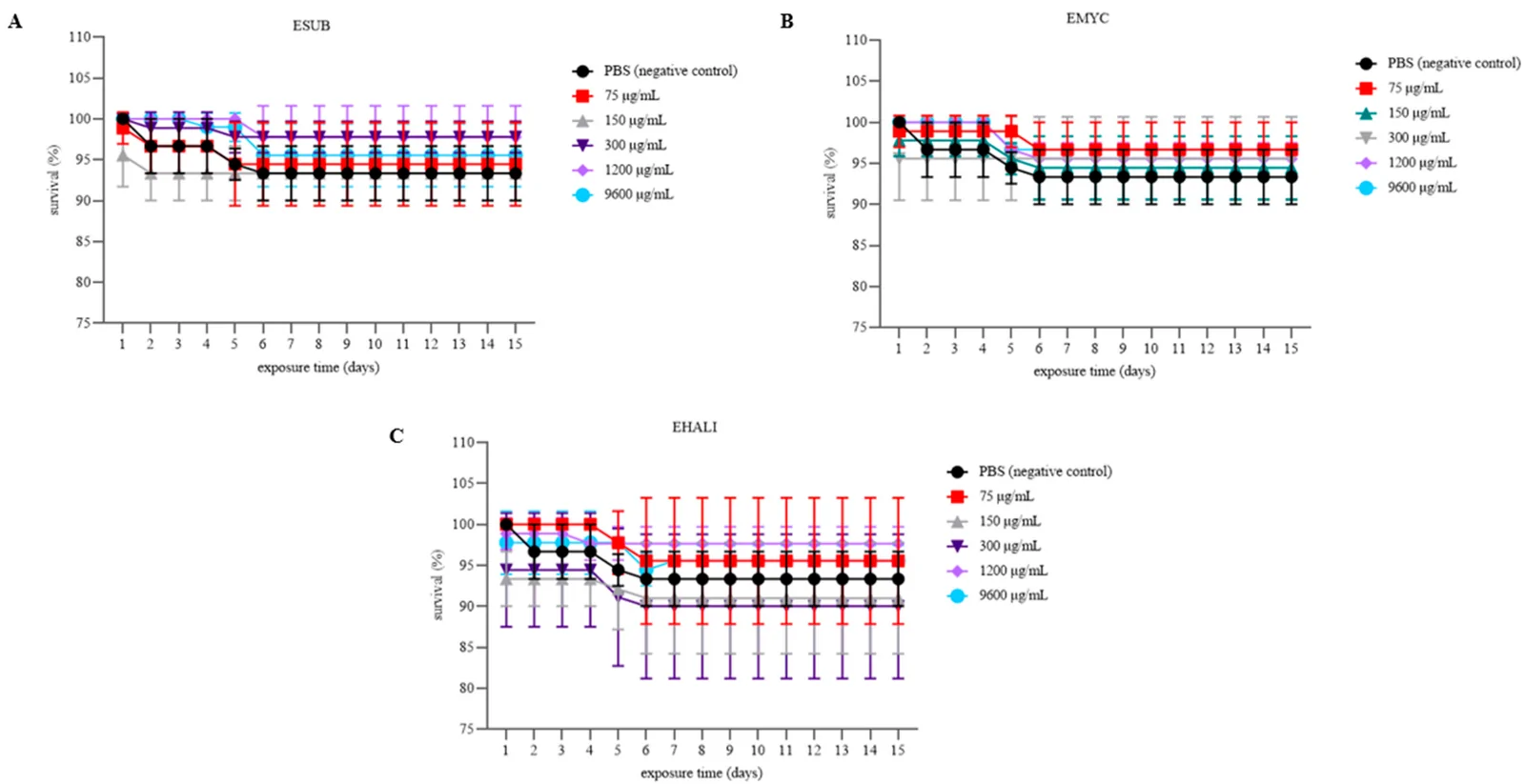
Survival percentage of larvae (*n* = 30 per group) inoculated with ESUB (**A**), EMYC (**B**), and EHALI (**C**) at different concentrations, compared with the negative control (PBS), evaluated over a period of 15 days. Values are expressed as the mean of triplicates ± standard deviation.

Toxicity was further evaluated over a 15-day period in larvae of *Tenebrio molitor* following inoculation with the extracts, compared with the negative control consisting of larvae injected with Phosphate-buffered saline (Figure 5).

Overall, none of the extracts exhibited detectable toxicity in *T. molitor* larvae, as no statistically significant differences were observed relative to the negative control group (larvae inoculated with PBS).

The table below summarizes the results obtained from the antioxidant and toxicological assays. Notably, EHALI demonstrated the highest antioxidant potential when compared with the other two extracts (Table 8).

## 3. Discussion

In the present study, extracts obtained from the aforementioned marine sponges were prepared using a methanol–chloroform mixture (1:1). The use of a combination of two solvents in this study ensured efficient extraction of all total lipid fractions (polar and neutral), proving more effective than the exclusive use of n-hexane.

Approximately 50% of the compounds were shared among the three extracts, including 1-dodecanol, dodecyl acrylate, hexadecanoic acid 2-hydroxy-1-(hydroxymethyl)ethyl ester, n-hexadecanoic acid, palmitic acid methyl ester, propanoic acid 3-mercapto- dodecyl ester, dl-chimyl alcohol, and cholesterol. The extracts EHALI and EMYC shared hydrocinnamic acid, whereas EHALI and ESUB shared γ-sitosterol, and ESUB and EMYC shared batyl alcohol, 1-hexadecanol, and palmitoleic acid.

Regarding compounds unique to each species, EMYC contained benzeneacetic acid, 2-piperidinone, and cholestan-3-ol, while ESUB contained 1-octadecanol, cholestanol, ergostanol, methyl stearate, and phenol 2,2′-methylenebis[6-(1,1-dimethylethyl)-4-methyl-]. In contrast, EHALI contained octadecanoic acid, octadecanoic acid 2-hydroxy-1-(hydroxymethyl)ethyl ester, ergosta-5,22-dien-3-ol (3β,22E,24S), ergosta-5,24(28)-dien-3-ol (3β), and campesterol. Among these, ESUB exhibited a high proportion of amphipathic compounds, with cholestanol representing the predominant constituent.

When comparing the chemical profiles obtained in this study with those previously reported for the genera corresponding to the investigated sponge species, it becomes evident that the extracts display a chemical profile consistent with existing literature. Our findings agree with previously reported metabolites for the genus *Suberites*. For instance, fatty acid methyl esters and pyrrolidide derivatives have been reported from *Suberites aurantiacus* collected in Cuba [16]. Additionally, sesterterpenes such as suberitenones C and D, as well as suberiphenol, have been isolated from *Suberites* species [17], along with suberitenones A and B [18] and okadaic acid from *Suberites domuncula* [19]. These compounds largely belong to the lipid class, supporting the predominance of lipid-derived metabolites observed in our analysis.

Among the compounds identified in ESUB, only palmitoleic acid, hexadecanoic acid, and palmitic acid have previously been reported in the genus. Furthermore, for the species *S. aurantiacus*, this study represents the first report of cholestanol in the crude extract, as well as the first report of several other compounds identified herein.

Consistent with the results observed for ESUB, cholestanol has also been reported as the major component in other species of the genus. In *Suberites domuncula* collected in Italy, extraction with chloroform and methanol revealed a predominance of 5α-cholestan-3β-ol (56%), followed by 13 sterols in smaller proportions [20].

For members of the family *Mycalidae*, the review conducted by Habener, Hooper, and Carroll [21] reported that nearly half of the identified compounds are alkaloids, followed by polyketides, terpenoids, and lipids, particularly sterols and steroidal glycosides. Additional compounds reported from this family include peptides, nucleosides, and nucleobases.

Similarly, a wide diversity of lipid compounds has been reported in extracts obtained from marine sponges belonging to the order Halichondrida [22]. However, variations in lipid composition have been observed when these organisms are collected from environments with extreme temperature differences. In a study conducted by Blumenberg and Michaelis [23], five sponge species from the order Halichondrida collected in Norway exhibited a high predominance of brominated fatty acids among the approximately 90 fatty acids identified by GC–MS. The authors suggested that these brominated lipids may play a role in modifying sponge cell membranes as an adaptive mechanism to cold environments, whereas such compounds appear to be less prevalent in species inhabiting warmer waters.

The concentration of these metabolites may vary depending on environmental factors, including the location where the sponge was collected, seasonal variation, and consequently the temperature and light conditions to which the organism is exposed. Furthermore, the production of certain metabolites is likely induced when sponges experience stress conditions that require biochemical responses, such as physical damage or injury [24]. Our research involved collecting samples during the rainy season; therefore, sampling during the dry season may result in qualitative and quantitative differences in the metabolites present in marine sponges.

In the EHALI extract, the predominant compounds were ergosta-5,24(28)-dien-3-ol (3β) (19.33%) and cholesterol (17.56%), whereas in EMYC, cholestan-3-ol (27.68%), dodecyl acrylate (21.75%), and cholesterol (15.17%) represented the major constituents.

Among the compounds detected in our study, hydrocinnamic acid (present in EMYC) and phenol 2,2′-methylenebis[6-(1,1-dimethylethyl)-4-methyl-] (present in ESUB) belong to the class of phenolic compounds, which are widely recognized for their antioxidant properties in extracts from various organisms [25]. This activity is primarily attributed to the presence of a hydroxyl (–OH) group, which, according to Rice-Evans, Miller, and Paganga [26], is responsible for hydrogen donation or electron transfer during free radical neutralization. However, the absence of phenolic compounds in EHALI did not prevent its antioxidant activity; therefore, it is not possible to determine which compounds, from each extract, are responsible for the observed antioxidant activities.

In the present study, the ability of the extracts to scavenge the radical DPPH was evaluated, revealing a dose-dependent effect. However, at 5000 µg/mL, a decrease in activity was observed for ESUB and EHALI. One possible explanation for this phenomenon involves the increased presence of interfering compounds within the extract. As extract concentration increases, the effective interaction between antioxidant molecules and DPPH may be reduced due to competition with other biomolecules present in the extract that may also interact with the radical. This interpretation is consistent if the observed activity does not arise from a synergistic effect among compounds.

Beyond in vitro antioxidant assays, several studies have also investigated the in vivo antioxidant potential of natural extracts. Experiments using *Tenebrio molitor* larvae have proven effective for evaluating antioxidant molecules, since this insect produces reactive oxygen species under stress conditions [27]. In this context, Silva Cordeiro et al. [28] used Copper sulfate to induce oxidative stress in *T. molitor* larvae and demonstrated that extracts from leaves and fruit peels of *Talisia esculenta* (100 and 250 µg/mL) effectively protected the larvae. The authors associated both in vitro antioxidant activity (DPPH scavenging, copper chelation, and CAT assays) and in vivo protection with the presence of phenolic compounds and flavonoids in the plant extracts.

Although the DPPH scavenging activity reported in that study was higher than that observed in the present work, the copper-chelating assay in our study employed only one quarter of the sample concentration (25 µg/mL) used in their experiment. Under these conditions, the extracts ESUB (113.45% ± 1.64), EHALI (112.10% ± 1.93), and EMYC (110.6% ± 1.02) demonstrated substantially higher copper-chelating activity compared with the values reported for the plant extracts (approximately 60% activity at 100 µg/mL). Therefore, it can be inferred that this antioxidant activity may also persist in vivo, as demonstrated in the aforementioned study.

**Parte inferior do formulário**

Another study reporting similar activities evaluated 141 extracts obtained from 47 marine sponges collected in the Indian Ocean for their ability to scavenge DPPH and chelate iron ions. The extract of *Pseudosuberites* sp. showed the highest iron-chelating activity (10.57 ± 0.39 mM Fe^2+^/g extract), followed by *Mycale tenuispiculata* (6.36 ± 0.31 mM Fe^2+^/g extract). In contrast, *Axinella donnani* exhibited the highest DPPH scavenging activity (92.15 ± 0.09%) at 1000 µg/mL extract. *Mycale tenuispiculata* showed less than 10% DPPH scavenging, while *Pseudosuberites aff. andrewsi* showed approximately 50%, *Pseudosuberites* sp. 40%, and *Suberites* sp. 0%. The authors associated these activities with the presence of phenolic compounds in the extracts [29].

In our study, in the DPPH assay at the same concentration (1000 µg/mL), EHALI (60.91 ± 3.05%) showed the highest activity, followed by ESUB (59.32 ± 0.76%) and EMYC (53.78 ± 1.06%). However, at double this concentration, ESUB showed the highest activity (132.54 ± 0.91%), followed by EHALI (75.02 ± 3.81%) and EMYC (60.55 ± 0.76%). When comparing the EC_50_ values for each assay (0.05 mg/mL, 0.14 mg/mL, and 0.06 mg/mL for DPPH scavenging by ESUB, EMYC, and EHALI, respectively), we also found other studies reporting lower reducing potential at higher concentrations. For example, extracts from propolis wax and honey showed an EC_50_ of 28 mg/mL for approximately 40% DPPH reducing power, while extracts from the alga *Dictyota dichotoma* presented an EC_50_ of 0.12 mg/mL for approximately 90% reducing power, which was associated with phenolic compounds present in the extract [30].

Although our samples showed high activity in specific assays, when evaluating the Total Antioxidant Capacity (TAC) using the TAC method, this potential was significantly lower. The reduction values of Mo^6+^ to Mo^5+^ expressed as ascorbic acid equivalents per gram of sample were 2.2 Eq. AA/g, 3.6 Eq. AA/g, and 6.6 Eq. AA/g for ESUB, EBMY, and EHALI, respectively. The TAC assay is performed under acidic pH and high temperature (100 °C), which may have influenced the electron-donating capacity of the samples and affected their solubility. The presented dataset reveals the antioxidant activity of ESUB, EMYC, and EHALI using different methods: all extracts also demonstrated the ability to donate electrons or hydrogen atoms to stabilize free radicals (DPPH assay). In addition, they exhibited metal-chelating activity (Cu^2+^), an important mechanism for preventing ROS formation in metal-catalyzed reactions, and we thus considered a preventive antioxidant strategy. Our work expands current knowledge of the reducing behavior of these extracts, which had previously been unexplored, and highlights the importance of their use.

Regarding the toxicological assays, a slight increase in hemolytic activity was observed for the three extracts at the highest concentration tested (IC_50_ > 9.6 mg/mL), likely due to the interaction between amphipathic compounds and the erythrocyte membrane. However, this effect was not observed at the other concentrations. This result differs from the study conducted by Purushottama et al. [31], in which extracts obtained from *Halichondria panicea* at 5000 µg/mL showed hemolytic activity in human and chicken erythrocytes when extracted with chloroform–methanol or methanol alone, whereas the aqueous extract did not show this effect. Due to the extraction method, it can be inferred that apolar or amphipathic compounds were responsible for the hemolytic activity observed in the first two extracts.

The low preliminary toxicity in erythrocytes and in *Tenebrio molitor* larvae represents an important pillar for the biotechnological viability of these extracts in potential future industrial applications. Recent evidence suggests that these insects can be effectively used in acute toxicological studies prior to testing in mammals and conducting clinical trials [32]. However, future tests with vertebrates are needed to define toxicological safety. Furthermore, although the extracts do not exhibit hemolytic activity, with direct damage to the cell membrane, further tests can be conducted to evaluate their effects on metabolically active cells in order to better understand their potential impacts on cellular metabolism.

## 4. Materials and Methods

### 4.1. Marine Sponges

**Figure 6:**
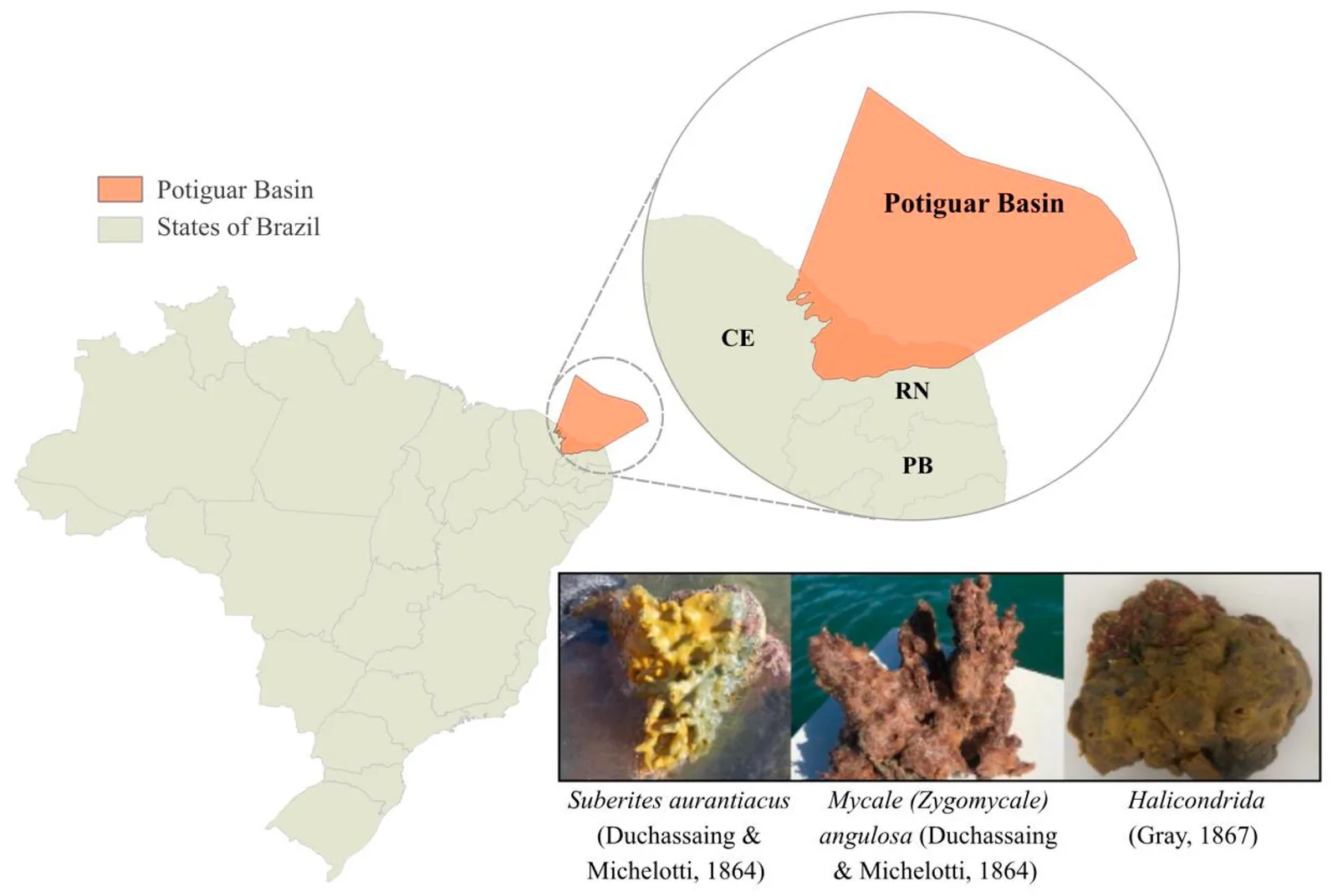
Potiguar Basin. Made by QGIS 3.4 software; Sponges used in this study.

Marine sponges were collected in the municipality of Macau during the rainy season by scuba diving at Praia de Camapum and in saline tanks near the beach, in collaboration with José Garcia and João Vitor Campelo Rodrigues. The collected material was stored on ice, and specimens fixed in 80% ethanol were sent to the Federal University of Ceará (Fortaleza, Brazil) for identification. Sponge identification was performed through spicule analysis by Sula Salani, who assigned the following voucher numbers for each identified sponge: POR-346—*Mycale (Zygomycale) angulosa* (Duchassaing & Michelotti, 1864); POR-357—*Suberites aurantiacus* (Duchassaing & Michelotti, 1864); and POR-366—Halichondrida (Figure 6).

### 4.2. Extraction of Biomolecules

**Figure 7:**
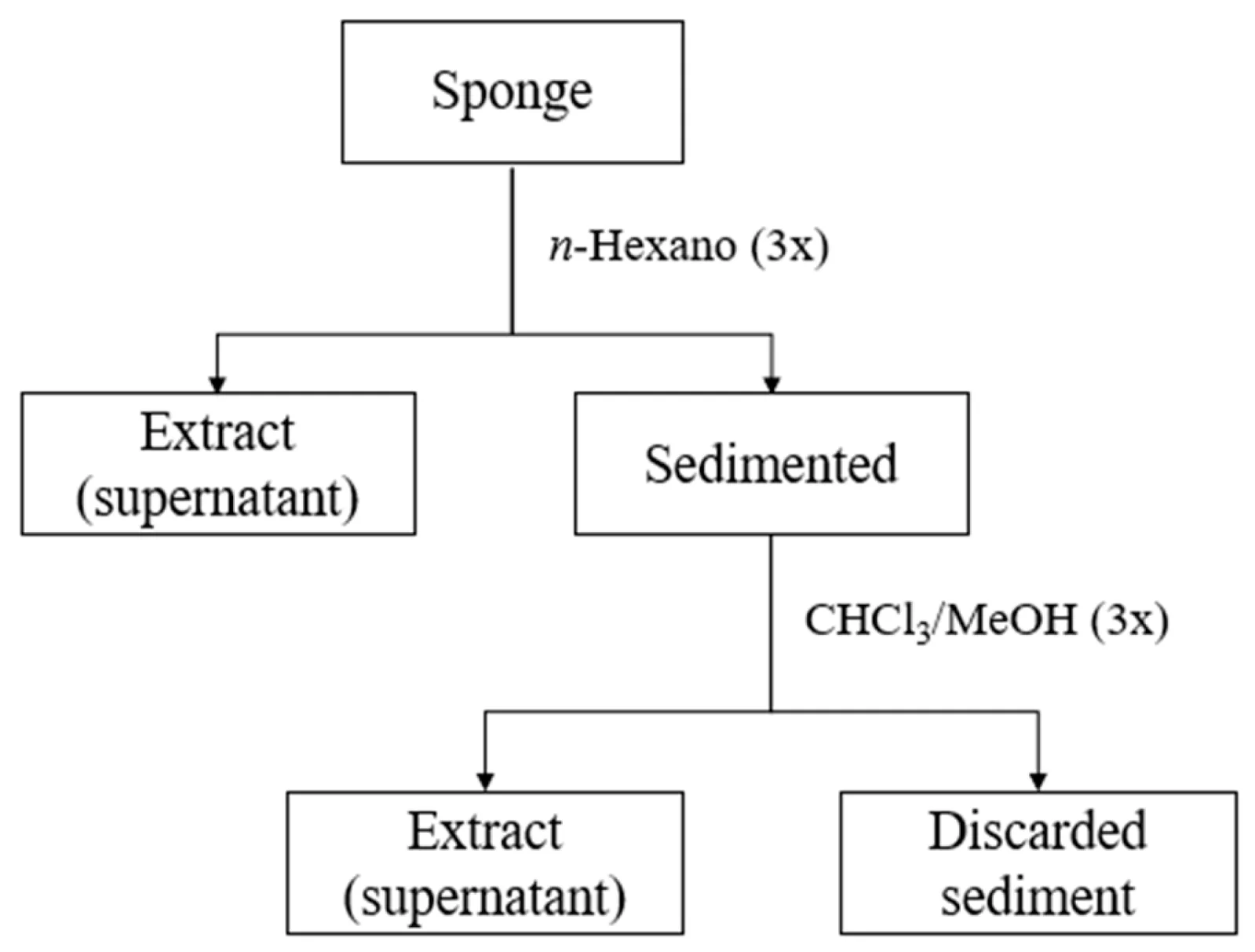
Flowchart of the biomolecule extraction steps.

The sponges, frozen during collection, were washed with distilled water to remove sediments and encrusting organisms and subsequently fragmented into small pieces. A 200 g sample of the whole sponge was separated. A preliminary extraction was then performed to remove the most hydrophobic fraction of the compounds using 300 mL of analytical-grade *n*-hexane (1:1) for 24 h; this procedure was repeated two additional times (totaling 900 mL of extraction solvent). The soluble fraction (extract) was dried in a rotary evaporator at 35 °C, and the remaining sediment was re-extracted with chloroform and methanol (1:1), as previously described. The soluble fraction was then dried, and the remaining sediment was discarded (Figure 7). After removal from the rotary evaporator, the extracts were lyophilized to eliminate any residual solvent or water. The obtained extracts were resuspended in PBS buffer (150 mM, pH 7) and stored at −20 °C for subsequent experiments.

### 4.3. Characterization of the Chemical Profile of the Extracts

To investigate the chemical profile of the chloroform/methanol extracts from the three sponges, analyses were performed using Gas–Liquid Chromatography–Mass Spectrometry (Shimadzu, Kyoto, Japão).

For sample preparation, the three extracts were diluted in 1.0 mL of HPLC-grade methanol and placed in an ultrasonic bath for 30 min. After extraction, the samples were filtered through a 0.22 µm PTFE syringe filter and transferred directly to vials. Sample analysis was carried out using a GC-2010-PLUS chromatograph coupled to a Shimadzu quadrupole mass spectrometer (GC–MS) and equipped with an automatic sampler. Separation of compounds was performed on an SH-Rtx-5MS column (30 m × 0.25 mm I.D., 0.25 μm film thickness). A volume of 1.0 µL was injected in splitless mode with a helium flow rate of 1 mL/min. Operational temperatures were set as follows: ion source at 230 °C, interface at 280 °C, and injector at 250 °C. The mass scan range was configured between *m/z* 50 and 500. Data acquisition started 5 min after the beginning of the run. The oven temperature was initially maintained at 75 °C for the first 5 min and then gradually increased to 290 °C at a rate of 6 °C per minute, remaining at this temperature for 20 min. The total analysis time was 60 min. Compounds were identified based on their retention times and by comparison with the National Institute of Standards and Technology spectral library.

### 4.4. Antioxidant Potential

#### 4.4.1. Cupric Ion Chelating Activity (Cu^2+^)

To evaluate the copper-chelating capacity of the samples, the reagent Pyrocatechol Violet (Sigma, St. Louis, MO, USA) was used because of its ability to bind to cations such as copper and detect their presence in assays. Test samples were evaluated at different concentrations (25, 50, 75, and 1000 µg/mL). For the microplate assay, 6 µL of pyrocatechol violet (4 mM) were added to the samples. Subsequently, 100 µL of Copper(II) sulfate pentahydrate were added, and the wells were homogenized before reading at 632 nm. Ethylenediaminetetraacetic acid was used as the standard, and the results were expressed as the percentage of copper ion chelation activity [33].

#### 4.4.2. DPPH Radical Scavenging

To evaluate the electron-donating activity toward the free radical DPPH, test samples were assessed for their ability to inhibit the formation of a purple-blue complex. The samples were evaluated at different concentrations (250, 500, 1000, and 2000 µg/mL) with 200 µL of an ethanolic DPPH solution (150 µM) and incubated at room temperature for 30 min. Absorbance was then measured at 517 nm, and the results were expressed as the percentage of DPPH scavenging activity.

#### 4.4.3. Total Antioxidant Capacity Assay (TAC)

Samples (100 and 1000 µg/mL) were incubated with a reagent solution containing sodium phosphate (28 mM), sulfuric acid (0.6 M), and ammonium molybdate (4 mM) initially at room temperature. The mixture was then incubated in an oven at 100 °C for 90 min, and absorbance was measured at 695 nm using a microplate reader [34].

### 4.5. Toxicological Tests

#### 4.5.1. Hemolytic Activity

To evaluate the hemolytic activity of the samples, blood collected in tubes containing Ethylenediaminetetraacetic acid was washed five times with 0.9% (*w*/*v*) saline solution (NaCl) through successive centrifugations at 924× *g* for 5 min at room temperature to remove plasma. This procedure ensured the isolation of intact erythrocytes, whose concentration was adjusted to 1% using the microhematocrit technique. For the assay, 100 µL of the 1% erythrocyte suspension were incubated in test tubes with 100 µL of the extracts at concentrations of 75, 150, 300, 1200, and 9600 µg/mL for 1 h at room temperature. For the positive control corresponding to 100% hemolysis, 100 µL of the erythrocyte suspension were mixed with 100 µL of 1% Triton X-100 (*v*/*v*). A mixture of 100 µL of the 1% erythrocyte suspension and 100 µL of PBS (150 mM, pH 7) was used as the reference for 0% hemolysis. After incubation, the tubes were centrifuged at 8000× *g* for 3 min, and 100 µL aliquots of the supernatants were transferred to a 96-well microplate and analyzed at 405 nm using a microplate reader. Hemolytic activity was calculated using the following formula:

(%) = [(Aa − Ab) × 100]/(Ac − Ab)

where Aa is the absorbance of the sample; Ab is the absorbance of the negative control; and Ac is the absorbance of the positive control.

#### 4.5.2. Toxicity in *Tenebrio molitor* Larvae

For the assays, larvae with a moderately developed size (weighing 100–150 mg) were used, with 30 larvae per group (tests performed in triplicate). Each group was divided into three separate Petri dishes to allow better accommodation. The larvae were kept protected from light and without food. A volume of 5 μL of the samples (at concentrations of 75, 150, 300, 1200, and 9600 µg/mL) was inoculated into the ventral portion of the larval hemocoel, between the second and third abdominal segments. After a period of 2 h, 5 μL of PBS were inoculated. For the negative control, 5 μL of PBS were inoculated, followed by an additional 5 μL of PBS after 2 h. Inoculations were performed using a Hamilton 10 µL microsyringe. During a period of 15 days, survival curves were constructed for each group. Larvae were considered dead when they showed no movement in response to mechanical stimulation (gentle pressure with forceps) and exhibited visible melanization of the cuticle.

### 4.6. Statistical Analysis

All tests were performed in triplicate using independent batches, and the results are presented as the mean with their respective standard deviation. The software GraphPad Prism, version 9.0, was used for data analysis. To determine statistical differences, the data were compared using Analysis of Variance followed by Tukey’s test and Dunnett’s test.

## 5. Conclusions

The present study expands the chemical knowledge of three species of marine sponges collected in a coastal region of northeastern Brazil that remains largely unexplored for this purpose. The results suggest that chloroform–methanol extracts obtained from the marine sponges *Suberites aurantiacus*, *Mycale (Zygomycale) angulosa*, and Halichondrida collected in the Potiguar Basin may present different metabolites depending on the collection site. While some metabolites found in the extracts of these three species had been previously reported in marine sponges from the same genus, our study identified known metabolites that had not yet been documented in these particular species. However, sampling sponges at different times of the year may result in qualitative and quantitative differences in the metabolites present. These metabolites may therefore be isolated and investigated in future studies to evaluate their bioactivity. Furthermore, the compounds present in the three extracts exhibited antioxidant activity through different methods and showed low toxicity in vitro and in vivo, indicating that they may represent promising sources for treatments aimed at oxidative stress control. Our results indicate that the Potiguar Basin is underexplored, and the data presented on chemical and biological diversity are unprecedented in the region, highlighting its relevance to the marine biodiversity of Latin America and its biotechnological potential, which is poorly documented internationally.

## Figures and Tables

**Table 1:** Yield of dry extract (in grams) obtained from each sponge sample.

Sponges	Extracts with *n*-hexane	Extracts with CHCl_3_ and MeOH
*S. aurantiacus*	0.063 g	3.63 g
*M. angulosa*	0.059 g	2.88 g
Halichondrida	0.042 g	1.67 g

**Table 2:** Identification of compounds present in ESUB by gas–liquid chromatography coupled with mass spectrometry (GC–MS) and their respective percentage of sample peak area.

RT (min)	Área (Counts·s)	Área (%)	Substance (NIST)
18.237	5,835,354.858	3.631	1-Dodecanol
22.533	13,396,437.827	8.336	Dodecyl acrylate
24.783	626,761.122	0.390	N.I.
25.789	1,707,507.597	1.062	1-Hexadecanol
26.549	1,056,733.521	0.658	Palmitic acid, methyl ester
26.884	1,846,305.775	1.149	Palmitoleic acid
26.991	1,245,937.594	0.775	N.I.
27.183	1,562,956.872	0.973	n-Hexadecanoic acid
27.966	1,128,314.198	0.702	Propanoic acid, 3-mercapto-, dodecyl ester
28.767	1,114,298.818	0.693	N.I.
29.041	1,163,482.716	0.724	1-Octadecanol
29.698	778,418.843	0.484	Methyl stearate
33.538	2,948,652.424	1.835	dl-Chimyl alcohol
33.915	1,218,763.284	0.758	Phenol, 2,2′-methylenebis[6-(1,1-dimethylethyl)-4-methyl-
34.399	684,785.641	0.426	N.I.
34.533	1,882,488.814	1.171	Batilol
34.895	524,173.965	0.326	N.I.
35.025	2,760,863.268	1.718	Hexadecanoic acid, 2-hydroxy-1-(hydroxymethyl)ethyl ester
36.209	1,629,198.652	1.014	N.I.
37.590	822,891.041	0.512	N.I.
39.605	1,851,184.930	1.152	N.I.
42.366	1,801,983.514	1.121	Cholesterol
42.545	101,946,682.834	63.436	Cholestanol
42.614	2,602,424.496	1.619	N.I.
42.946	674,770.349	0.420	N.I.
43.049	1,619,218.459	1.008	N.I.
44.008	3,286,330.752	2.045	Ergostanol
45.323	2,991,520.459	1.861	γ-Sitosterol

N.I.: Not identified; RT: Retention Time.

**Table 3:** Chemical classes of compounds identified in ESUB.

Compound	Chemical Class	Wm	Formula
**1-Dodecanol**	Fatty alcohol	186.33	C_12_H_26_O
**Dodecyl acrylate**	Fatty alcohol	240.38	C_15_H_28_O_2_
**1-Hexadecanol**	Fatty alcohol	242.44	C_16_H_34_O
**Palmitic acid, methyl ester**	Fatty acid	270.45	C_17_H_34_O_2_
**Palmitoleic acid**	Fatty acid	254.41	C_16_H_30_O_2_
**n-Hexadecanoic acid**	Fatty acid	256.42	C_16_H_32_O_2_
**Propanoic acid, 3-mercapto-, dodecyl ester**	Thiol ester	274.46	C_15_H_30_O_2_S
**1-Octadecanol**	Fatty alcohol	270.49	C_18_H_38_O
**Methyl stearate**	Fatty alcohol	298.50	C_19_H_38_O_2_
**dl-Chimyl alcohol**	Alkylglycerol	316.5	C_19_H_40_O_3_
**Phenol, 2,2′-methylenebis[6-** **(1,1-dimethylethyl)-4-methyl-**	Phenolic compound	340.50	C_23_H_32_O_2_
**Batilol**	Alkylglycerol	344.57	C_21_H_44_O_3_
**Hexadecanoic acid, 2-hydroxy-1-** **(hydroxymethyl)ethyl ester**	Monoglyceride	330.50	C_19_H_38_O_4_
**Cholesterol**	Sterol	386.65	C_27_H_46_O
**Cholestanol**	Sterol	388.67	C_27_H_48_O
**Ergostanol**	Sterol	402.7	C_28_H_50_O
**γ-Sitosterol**	Sterol	414.7	C_29_H_50_O

**Table 4:** Identification of compounds present in EMYC by gas–liquid chromatography coupled with mass spectrometry (GC–MS) and their respective percentage of sample peak area.

RT (min)	Area (Counts·s)	Área (%)	Substance (NIST)
11.448	2,237,681.256	2.676	2-Piperidinone
13.452	1,445,313.521	1.728	Benzeneacetic acid
15.420	1,449,483.679	1.733	Hydrocinnamic acid
18.242	7,644,848.123	9.141	1-Dodecanol
22.541	18,190,781.235	21.750	Dodecyl acrylate
24.783	529,963.669	0.634	N.I.
25.791	528,207.808	0.632	1-Hexadecanol
26.549	748,594.606	0.895	Palmitic acid, methyl ester
26.872	501,938.819	0.600	Palmitoleic acid
27.188	1,340,217.216	1.602	n-Hexadecanoic acid
27.969	1,468,616.646	1.756	Propanoic acid, 3-mercapto-, dodecyl ester
33.546	4,564,422.396	5.458	dl-Chimyl alcohol
34.532	755,039.697	0.903	Batilol
35.025	2,319,195.933	2.773	Hexadecanoic acid, 2-hydroxy-1-(hydroxymethyl)ethyl ester
36.211	783,613.875	0.937	N.I.
37.592	714,280.281	0.854	N.I.
39.603	869,672.021	1.040	N.I.
42.354	12,693,374.368	15.177	Cholesterol
42.482	23,155,403.372	27.686	Cholestan-3-ol
43.052	1,694,306.915	2.026	N.I.

N.I.: Not identified; RT: Retention Time.

**Table 5:** Chemical classes of compounds identified in EMYC.

Compound	Chemical Class	Wm	Formula
**2-Piperidinone**	Alkaloid	99.13	C_5_H_9_NO
**Benzeneacetic acid**	Monocarboxylic acid	136.15	C_8_H_8_O_2_
**Hydrocinnamic acid**	Monocarboxylic acid	150.17	C_9_H_10_O_2_
**1-Dodecanol**	Fatty alcohol	186.33	C_12_H_26_O
**Dodecyl acrylate**	Fatty alcohol	240.38	C_15_H_28_O_2_
**1-Hexadecanol**	Fatty alcohol	242.44	C_16_H_34_O
**Palmitic acid,** **methyl ester**	Fatty acid	270.45	C_17_H_34_O_2_
**Palmitoleic acid**	Fatty acid	254.41	C_16_H_30_O_2_
**n-Hexadecanoic acid**	Fatty acid	256.42	C_16_H_32_O_2_
**Propanoic acid, 3-mercapto-, dodecyl ester**	Ester	274.46	C_15_H_30_O_2_S
**dl-Chimyl alcohol**	Alkylglycerol	316.5	C_19_H_40_O_3_
**Batilol**	Alkylglycerol	344.57	C_21_H_44_O_3_
**Hexadecanoic acid, 2-hydroxy-1-(hydroxymethyl)ethyl ester**	Monoglyceride	330.50	C_19_H_38_O_4_
**Cholesterol**	Sterol	386.65	C_27_H_46_O
**Cholestan-3-ol**	Sterol	388.67	C_27_H_48_O

**Table 6:** Identification of compounds present in EHALI by gas–liquid chromatography coupled with mass spectrometry (GC–MS) and their respective percentage of sample peak area.

RT (min)	Area (Counts·s)	Área (%)	Substance (NIST)
15.366	737,105.182	0.914	Hydrocinnamic acid
18.234	5,938,271.368	7.363	1-Dodecanol
22.524	6,858,333.224	8.503	Dodecyl acrylate
26.547	739,457.114	0.917	Palmitic acid, methyl ester
27.191	2,196,688.581	2.724	n-Hexadecanoic acid
27.963	836,320.036	1.037	Propanoic acid, 3-mercapto-, dodecyl ester
30.264	859,928.116	1.066	Octadecanoic acid
33.532	1,336,987.999	1.658	dl-Chimyl alcohol
35.027	4,015,567.970	4.979	Hexadecanoic acid, 2-hydroxy-1-(hydroxymethyl)ethyl ester
36.212	2,242,498.351	2.780	N.I.
37.588	1,384,824.075	1.717	Octadecanoic acid, 2-hydroxy-1-(hydroxymethyl)ethyl ester
39.601	1,653,110.100	2.050	N.I.
41.847	3,541,613.677	4.391	N.I.
42.343	14,163,123.247	17.560	Cholesterol
42.448	879,562.304	1.091	N.I.
42.952	6,373,288.159	7.902	Ergosta-5,22-dien-3-ol, (3β,22E,24S)-
43.792	15,590,401.188	19.330	Ergosta-5,24(28)-dien-3-ol, (3β)-
43.869	442,5961.728	5.487	Campesterol
44.376	1,110,068.549	1.376	N.I.
45.329	5,155,988.066	6.393	γ-Sitosterol
45.645	616,500.323	0.764	N.I.

N.I.: Not identified; RT: Retention Time.

**Table 7:** Chemical classes of compounds identified in EHALI.

Compound	Chemical Class	WM	Formula
**Hydrocinnamic acid**	Phenolic compound	150.17	C_9_H_10_O_2_
**1-Dodecanol**	Fatty alcohol	186.33	C_12_H_26_O
**Dodecyl acrylate**	Fatty alcohol	240.38	C_15_H_28_O_2_
**Palmitic acid,** **methyl ester**	Fatty acid	270.45	C_17_H_34_O_2_
**n-Hexadecanoic acid**	Fatty acid	256.42	C_16_H_32_O_2_
**Propanoic acid, 3-mercapto-, dodecyl ester**	Ester	274.46	C_15_H_30_O_2_S
**Octadecanoic acid**	Fatty acid	284.47	C_18_H_36_O_2_
**dl-Chimyl alcohol**	Alkylglycerol	316.5	C_19_H_40_O_3_
**Hexadecanoic acid, 2-hydroxy-1-(hydroxymethyl)ethyl ester**	Monoglyceride	330.50	C_19_H_38_O_4_
**Octadecanoic acid, 2-hydroxy-1-(hydroxymethyl)ethyl ester**	Fatty acid	330.50	C_21_H_42_O_4_
**Cholesterol**	Sterol	386.65	C_27_H_46_O
**Ergosta-5,22-dien-3-ol, (3β,22E,24S)-**	Sterol	386.65	C_28_H_46_O
**Ergosta-5,24(28)-dien-3-ol, (3β)-**	Sterol	398.66	C_28_H_46_O
**Campesterol**	Sterol	400.7	C_28_H_48_O
**γ-Sitosterol**	Sterol	414.7	C_29_H_50_O

**Table 8:** Summary of antioxidant and toxicological assay results.

Activity	Samples		
	ESUB	EMYC	EHALI
TAC	2.2 Eq. AA/g ^a^	3.6 Eq. AA/g ^b^	6.6 Eq. AA/g ^c^
DPPH scavenging (EC_50_)	0.05 mg/mL ^a^	0.14 mg/mL ^b^	0.06 mg/mL ^a^
Copper chelating (EC_50_)	0.03 mg/mL ^a^	0.034 mg/mL ^b^	0.026 mg/mL ^c^
Hemolytic activity (IC_50_)	>9.6 mg/mL ^a^	>9.6 mg/mL ^a^	>9.6 mg/mL ^a^
In vivo toxicity (IC_50_)	>9.6 mg/mL ^a^	>9.6 mg/mL ^a^	>9.6 mg/mL ^a^

Eq. AA/g: ascorbic acid equivalents per gram of sample. Sample concentrations within each group were compared with one another. Letters “a”, “b” and “c” indicate statistically significant differences (*p* < 0.0001).

## Data Availability

The original contributions presented the study are included in the article. Further inquiries can be directed to the corresponding author.
